# The Effects of Intermittent Theta Burst Stimulation on Functional Brain Network Following Stroke: An Electroencephalography Study

**DOI:** 10.3389/fnins.2021.755709

**Published:** 2021-10-22

**Authors:** Qian Ding, Shunxi Zhang, Songbin Chen, Jixiang Chen, Xiaotong Li, Junhui Chen, Yuan Peng, Yujie Chen, Kang Chen, Guiyuan Cai, Guangqing Xu, Yue Lan

**Affiliations:** ^1^Department of Rehabilitation Medicine, Guangzhou First People’s Hospital, School of Medicine, South China University of Technology, Guangzhou, China; ^2^Department of Rehabilitation Medicine, Guangdong Provincial People’s Hospital, Guangdong Academy of Medical Sciences, Guangzhou, China

**Keywords:** intermittent theta burst stimulation (iTBS), electroencephalography (EEG), stroke, functional connectivity, graph theory

## Abstract

**Objective:** Intermittent theta burst stimulation (iTBS) is a special form of repetitive transcranial magnetic stimulation (rTMS), which effectively increases cortical excitability and has been widely used as a neural modulation approach in stroke rehabilitation. As effects of iTBS are typically investigated by motor evoked potentials, how iTBS influences functional brain network following stroke remains unclear. Resting-state electroencephalography (EEG) has been suggested to be a sensitive measure for evaluating effects of rTMS on brain functional activity and network. Here, we used resting-state EEG to investigate the effects of iTBS on functional brain network in stroke survivors.

**Methods:** We studied thirty stroke survivors (age: 63.1 ± 12.1 years; chronicity: 4.0 ± 3.8 months; UE FMA: 26.6 ± 19.4/66) with upper limb motor dysfunction. Stroke survivors were randomly divided into two groups receiving either Active or Sham iTBS over the ipsilesional primary motor cortex. Resting-state EEG was recorded at baseline and immediately after iTBS to assess the effects of iTBS on functional brain network.

**Results:** Delta and theta bands interhemispheric functional connectivity were significantly increased after Active iTBS (*P* = 0.038 and 0.011, respectively), but were not significantly changed after Sham iTBS (*P* = 0.327 and 0.342, respectively). Delta and beta bands global efficiency were also significantly increased after Active iTBS (*P* = 0.013 and 0.0003, respectively), but not after Sham iTBS (*P* = 0.586 and 0.954, respectively).

**Conclusion:** This is the first study that used EEG to investigate the acute neuroplastic changes after iTBS following stroke. Our findings for the first time provide evidence that iTBS modulates brain network functioning in stroke survivors. Acute increase in interhemispheric functional connectivity and global efficiency after iTBS suggest that iTBS has the potential to normalize brain network functioning following stroke, which can be utilized in stroke rehabilitation.

## Introduction

Stroke is one of the main causes of adult disability worldwide (Hankey, 2013). Upper extremity motor impairment is a common clinical representation following stroke. More than half of individuals experience upper extremity motor impairment acutely after stroke, and the motor deficits persist to the chronic phase in approximately two thirds of stroke survivors who initially had upper extremity motor impairment (Kwakkel et al., 2004; Tedesco Triccas et al., 2019). The persistent motor deficits following stroke may result from altered cortical activity and brain network functioning (Desowska and Turner, 2019; Vecchio et al., 2019). As one of the non-invasive brain stimulation techniques, repetitive transcranial magnetic stimulation (rTMS) offers a chance to modulate cortical excitability and correct abnormal cortical activity following stroke (Suppa et al., 2016), which has been suggested to be a promising approach for stroke rehabilitation (Corti et al., 2012).

Intermittent theta burst stimulation (iTBS) is a specific form of rTMS that effectively elevates cortical excitability of the stimulated brain regions for at least 20 min (Huang et al., 2005). As iTBS employs a shorter stimulation period and a lower stimulation intensity compared with traditional rTMS, iTBS could be a good rTMS option in clinical practice (Talelli et al., 2007). Neural effects of iTBS are typically investigated by motor evoked potentials (MEP), which are muscular responses elicited by single-pulse TMS (Huang et al., 2005; Talelli et al., 2007; Di Lazzaro et al., 2008; Ackerley et al., 2010; Hinder et al., 2014; Ding et al., 2021b). However, this approach is not applicable to stroke survivors in whom MEPs in the paretic limb cannot be elicited. In addition, it has been suggested that iTBS has impact on functional brain network in remote regions from the stimulated site (Suppa et al., 2016). As MEPs only reflect corticospinal excitability of primary motor cortex (M1), other neuroimaging tools are needed to complement with MEPs and investigate neurophysiological effects induced by iTBS from other aspects.

Electroencephalography (EEG) is a neuroimaging approach that records cortical electrical activity along the scalp. Resting-state EEG has been suggested to be a sensitive measure for evaluating effects of rTMS on brain functional activity (e.g., functional connectivity) (Casarotto et al., 2010). Functional connectivity refers to synchrony of cortical activity in anatomically distinct but functionally collaborating brain regions (Vecchio et al., 2019), which forms the basis of functional brain network. Graph theory analysis is an approach for characterizing functional brain network (Park et al., 2014). Based on graph theory, the average of interregional efficiency between every pair of brain region over the entire brain is called global efficiency, which measures the efficiency in transporting information at a global scale (Park et al., 2014). EEG-based functional brain network analysis could provide additional valuable information on the neural effects induced by iTBS.

Following stroke, focal brain lesions could cause alteration in the dynamics of functional brain network, which involves not only the damaged brain areas but also extending to remote areas (Vecchio et al., 2019). It has been reported that interhemispheric functional connectivity was reduced acutely after stroke, and increased gradually in parallel with motor improvements in stroke survivors, indicating a supportive role of interhemispheric functional connectivity in motor recovery following stroke (Golestani et al., 2013; Desowska and Turner, 2019; Hoshino et al., 2020; Li et al., 2020). Global efficiency has also been suggested to be reduced following stroke, and individuals with worse motor performance tend to have lower global efficiency (Philips et al., 2017). Therefore, brain network functioning can be considered as a potential biomarker indicating stroke recovery and has been frequently used as an outcome assessment in stroke studies (Caliandro et al., 2017; Philips et al., 2017; Vecchio et al., 2019). However, to our knowledge, no published study has applied EEG to evaluate the aftereffects of rTMS (including iTBS) on the functional brain network in stroke survivors.

van Meer et al. (2010) reported that impaired motor function acutely after experimental stroke in rats was related to partial loss of interhemispheric functional connectivity, and interhemispheric functional connectivity was increased subsequently concomitant to motor recovery. In humans, reduced interhemispheric functional connectivity was also observed acutely after stroke (Philips et al., 2017; Desowska and Turner, 2019; Hoshino et al., 2020; Li et al., 2020). It has been reported that the increase in interhemispheric functional connectivity was associated with motor improvements in stroke survivors, and restoration of interhemispheric functional connectivity was noted only in well recovered individuals, but not in the poorly recovered stroke survivors (Golestani et al., 2013; Desowska and Turner, 2019; Hoshino et al., 2020; Li et al., 2020), suggesting that interhemispheric functional connectivity is possibly a potential biomarker indicating stroke recovery (Caliandro et al., 2017; Philips et al., 2017; Vecchio et al., 2019).

The effects of iTBS or high frequency rTMS on functional brain network have been previously investigated in healthy adults (Nettekoven et al., 2014; Park et al., 2014; Hoy et al., 2016). Interhemispheric functional connectivity has been reported to be increased after iTBS in both EEG (Hoy et al., 2016) and functional magnetic resonance imaging (fMRI) (Nettekoven et al., 2014) studies. Park et al. (2014) used resting-state EEG to investigate the effects of high frequency rTMS on global efficiency in healthy adults, and an increase in global efficiency was observed in individuals with behavioral facilitation after rTMS. Due to the differences between healthy adults and stroke survivors, it is still unclear whether iTBS would produce similar effects on interhemispheric functional connectivity and global efficiency in stroke survivors.

In present study, we used resting-state EEG to investigate the effects of iTBS on functional brain network in stroke survivors. We anticipated that interhemispheric functional connectivity and global efficiency would be increased after iTBS. These results would have potential implications for understanding the influences of iTBS on functional brain network in stroke survivors.

## Materials and Methods

### Participants

Thirty stroke survivors participated in this study. Stroke survivors were included into this study if they had a single stroke less than 18 months prior to enrollment. All stroke survivors were screened for eligibility to receive iTBS and excluded if they were using medications that reduce seizure threshold or had history of seizure disorder; pregnant; or any implanted devices or metal that might be affected by iTBS (Rossi et al., 2009). Stroke survivors were also excluded if there was a presence of cognitive impairment as defined by inability to comprehend and follow three step commands (Ding et al., 2018). Upper-extremity component of the Fugl-Meyer motor function assessment (FMA) and action research arm test (ARAT) were used to assess motor impairment and upper extremity motor performance, respectively. Demographic characteristics are reported in Tables 1, 2.

**TABLE 1:** Patients’ demographic and clinical characteristics.

	**Age, years**	**Sex**	**Paretic side**	**Type of stroke**	**Months after stroke onset**	**UE FMA (0–66)**	**ARAT (0–57)**

	**Mean ± SD (range)**	**Male/Female**	**Right/Left**	**Ischemic/Hemorrhagic**	**Mean ± SD (range)**	**Mean ± SD (range)**	**Mean ± SD (range)**
Active iTBS group (*N* = 15)	65.1 ± 11.9 (35–85)	12/3	5/10	12/3	3.9 ± 3.0 (1–11)	28.0 ± 19.8 (4–62)	26.1 ± 20.9 (0–56)
Sham iTBS group (*N* = 15)	61.1 ± 12.1 (35–79)	9/6	7/8	12/3	4.0 ± 4.4 (1–18)	25.1 ± 18.8 (4–64)	21.8 ± 22.2 (0–57)

*UE FMA refers to upper-extremity component of the Fugl-Meyer Motor Function Assessment, indicating motor impairments in stroke survivors (Fugl-Meyer et al., 1975). ARAT refers to Action Research Arm Test, indicating upper extremity performance (i.e., coordination and dexterity) in neurological populations (Lang et al., 2006). SD refers to standard deviation. No significant difference in chronicity, UE FMA, or ARAT was revealed between subjects in Active and Sham iTBS groups.*

**TABLE 2:** Stroke characteristics.

**Subject number**	**Sex**	**Age (years)**	**Paretic hand**	**Chronicity (months)**	**Type of stroke**	**Lesion location**	**UE FMA**	**RMT (%MSO)**
Stroke 01	M	62	R	4	Ischemic	Basal ganglia	10	100
Stroke 02	M	70	L	8	Ischemic	Pons	37	70
Stroke 03	M	57	R	8	Hemorrhagic	Frontal/parietal lobe	47	55
Stroke 04	M	70	L	2	Ischemic	Basal ganglia, corona radiata	14	100
Stroke 05	F	85	L	1	Ischemic	Basal ganglia	62	55
Stroke 06	F	66	R	1	Ischemic	Frontal/temporal/parietal lobe	4	100
Stroke 07	M	77	L	1	Ischemic	Centrum semiovale, corona radiata, basal ganglia	58	70
Stroke 08	F	35	L	5	Ischemic	Frontal/parietal lobe	49	100
Stroke 09	M	69	R	11	Ischemic	Corona radiata	18	100
Stroke 10	M	46	L	5	Hemorrhagic	Posterior horn of lateral ventricle	27	100
Stroke 11	M	72	R	1	Hemorrhagic	Parietal/temporal lobe	47	40
Stroke 12	M	62	L	2	Ischemic	Pons	26	50
Stroke 13	M	63	L	6	Ischemic	Basal ganglia, corona radiata, centrum semiovale	12	100
Stroke 14	M	75	L	4	Ischemic	Pons	5	100
Stroke 15	M	67	L	2	Ischemic	Frontal/temporal/parietal lobe	4	100
Stroke C01	F	65	R	7	Ischemic	Basal ganglia, corona radiata	8	100
Stroke C02	M	69	R	2	Ischemic	Basal ganglia	28	80
Stroke C03	F	70	L	2	Ischemic	Basal ganglia, corona radiata, frontal lobe	45	100
Stroke C04	M	42	L	6	Hemorrhagic	Pons	21	100
Stroke C05	F	79	R	2	Ischemic	Thalamus/occipital lobe	36	40
Stroke C06	M	60	R	9	Ischemic	Frontal/parietal/temporal lobe	4	100
Stroke C07	M	43	L	1	Ischemic	Frontal/parietal lobe, basal ganglia, corona radiata	64	20
Stroke C08	M	67	L	2	Ischemic	Frontal/parietal/temporal lobe	4	100
Stroke C09	F	64	R	2	Hemorrhagic	Basal ganglia	12	50
Stroke C10	M	64	L	1	Ischemic	Corona radiata, basal ganglia, thalamus	49	78
Stroke C11	M	72	L	2	Ischemic	Basal ganglia, periventricular white matter	46	80
Stroke C12	F	64	R	3	Ischemic	Frontal/temporal/parietal lobe	4	100
Stroke C13	M	70	R	3	Ischemic	Basal ganglia	28	75
Stroke C14	M	35	L	18	Hemorrhagic	Basal ganglia	24	100
Stroke C15	F	52	L	1	Ischemic	Corona radiata	4	100

*UE FMA refers to upper-extremity component of the Fugl-Meyer Motor Function Assessment. M refers to male, and F refers to female. L refers to left, and R refers to right. RMT refers to resting motor thresholds in the ipsilesional hemisphere. MSO refers to maximum stimulator output. “RMT = 100” indicates that MEPs were unable to be elicited in the paretic hand. Stroke 1–15 indicate subjects in the Active iTBS group. Stroke C1-15 indicate subjects in the Sham iTBS group.*

Subjects gave their written informed consent for the experimental procedures that were approved by the Guangzhou First People’s Hospital Human Research Ethics Committee. The study was performed in accordance with the Declaration of Helsinki.

### Study Design

We used a sham-controlled, randomized single-blinded design. Stroke survivors were randomly assigned to the experimental (Active iTBS) and control (Sham iTBS) groups, with fifteen subjects in each group. Stroke survivors were blinded with respect to the group they were assigned to, that is, whether the subject received Active or Sham iTBS.

### Intermittent Theta Burst Stimulation

Intermittent theta burst stimulation was applied over the M1 in the ipsilesional hemisphere (IH) using a NS5000 Magnetic Stimulator (YIRUIDE Medical Co., Wuhan, China). The iTBS pattern consists of bursts containing three pulses at 50 Hz repeated at 5 Hz. A 2 s train of TBS was repeated every 10 s for a total of 192 s (600 pulses in total) (Huang et al., 2005). Of note, the stimulation intensity was set at 70% resting motor threshold (RMT) instead of 80% active motor threshold (AMT) in the original iTBS protocol (Volz et al., 2016). The reason for setting stimulation intensity based on RMT rather than AMT is that the latter would require stroke survivors to perform constant submaximal contractions of the target muscle which is often impossible for the paretic hand, especially in low-functioning stroke survivors (Volz et al., 2016). Furthermore, previous studies have shown similar aftereffects of iTBS with a stimulation intensity of 70% RMT or 80% AMT (Gentner et al., 2008; Cardenas-Morales et al., 2014). Therefore, a stimulation intensity of 70% RMT can be considered as an effective variant for increasing cortical excitability after iTBS (Volz et al., 2016; Yu et al., 2021).

Resting motor threshold determination was performed prior to the application of iTBS. Surface electromyography (EMG) was recorded from the first dorsal interosseous (FDI) in the paretic hand. Stroke survivors were seated in a comfortable chair with back support (Ding et al., 2018). TMS was applied over M1 using a figure-of-eight-shaped coil (70 mm diameter) positioned tangentially 45° from midline. Stroke survivors were asked to remain static while determining the optimal scalp position (i.e., “hotspot”) for eliciting maximal responses in the FDI (Ding et al., 2018). RMT was determined experimentally as the lowest stimulation intensity that produced MEP greater than 50 μV in at least 50% of consecutive stimulations at rest (Chen et al., 1998). A neuronavigation system (Visor2, ANT Neuro, Hengelo, Netherlands) was used to ensure reliable and consistent coil positioning over the “hotspot” throughout the experiment (Ding et al., 2021a).

Intermittent theta burst stimulation was applied over the “hotspot.” During the application of iTBS, stroke survivors were asked to remain static. As 40% maximum stimulator output (MSO) is the upper limit for iTBS with the NS5000 Magnetic Stimulator, stimulation intensity was set at 40% MSO for iTBS if the calculated stimulation intensity was greater than 40% MSO (i.e., for those whose RMT was greater than 57% MSO). For sham stimulation, the same stimulation intensity was used as for iTBS, and the TMS coil was held perpendicular to the skull, touching the skull with the rim opposite the handle (Nettekoven et al., 2014).

### Electroencephalography (EEG)

#### Electroencephalography Acquisition

Resting-state EEG was recorded at baseline and immediately after iTBS. During EEG recording, participants were seated comfortably in a sound-shielded, dimly lit room with eyes closed, which lasted for 6 min. EEG signals were recorded using a TMS-compatible EEG cap (ANT Neuro, Enschede, Netherlands) with 64 Ag/AgCl electrodes in a layout based on the extended international 10–20 system for electrodes placement (Jurcak et al., 2007; Tamburro et al., 2020). All channels were referenced online to CPz and amplified with an eego amplifier (ANT Neuro, Enschede, Netherlands). Data were sampled at 2,048 Hz with impedances kept below 10 kΩ for all channels throughout data collection.

#### Electroencephalography Analysis

Acquired EEG signals were analyzed off-line using MATLAB2019b (MathWorks, Inc., Natick, MA, United States). EEGLAB toolbox (version 14.1.2b) was used for EEG data preprocessing (Delorme and Makeig, 2004). After the raw data were imported into EEGLAB, the signals were sampled down to 1,000 Hz. Then, the EEG signals were filtered with a band-pass filter with cut-off values ranging from 0.1 to 40 Hz and segmented in epochs lasting 2,000 ms. The independent component analysis (ICA) was then performed to exclude components endowing eye (blink and movement), cardiac, and muscular artifacts. The resulting data was inspected to exclude remaining “bad trials” (i.e., amplitudes >100 μV) and re-referenced using the average signals of every scalp electrode as reference.

Power and functional connectivity analyses were conducted using customed MATLAB scripts. Absolute power (μV^2^) was calculated by fast Fourier transform and averaged in four frequency bands: delta (1–4 Hz), theta (4–8 Hz), alpha (8–13 Hz), beta (13–30 Hz). As we were interested in assessing cortical activity in bilateral sensorimotor cortices, the averaged power of the electrodes in the cluster of EEG electrodes around C3 and C4 (Left sensorimotor cortex: C1, C3, C5, CP1, CP3, CP5, FC1, FC3, FC5; Right sensorimotor cortex: C2, C4, C6, CP2, CP4, CP6, FC2, FC4, FC6) was calculated for statistical analysis (Bayram et al., 2015).

Coherence was calculated using customed MATLAB scripts to indicate functional connectivity between bilateral sensorimotor cortices. The Welch’s averaged, modified periodogram method (Welch, 1967), was performed to calculate the squared coherence between each pair of electrodes in four frequency bands. All connectivity matrices were Fisher’s z-transformed (Arun et al., 2020) to the set of Gaussian distributed values and the z scores were used for further analysis. As we were interested in assessing interhemispheric functional connectivity, the averaged z-scores of each pair of electrodes between sensorimotor cortices were calculated for statistical analysis.

GRaph thEoretical Network Analysis (GRETNA) toolbox was used for graph theory analysis (Wang et al., 2015). In general, a graph is based on a set of nodes. The connections between these nodes are edges, which form the brain network. In present study, weighted and undirected networks were built based on coherence (Vecchio et al., 2019). Since there was no definite method for selecting a single threshold, we integrated the metrics over the entire threshold range (i.e., 0.1–0.4, with an interval of 0.05) to obtain the area under the curve (AUC) to characterize the brain network (Wang et al., 2015; Yan et al., 2017). Global efficiency is the average of interregional efficiency between every pair of brain region over the entire brain, which characterizes information transferring ability in the entire brain network (G) (Park et al., 2014). It can be computed as the average of nodal efficiency across all nodes of the brain network:


$$E_{g\,l\,o\,b\,a\,l}\,\left(G\right)=\frac{1}{N\,\left(N-1\right)}\,\sum_{j\neq i\in G}\frac{1}{D\,\left(i,j\right)}$$

where D(i, j) is the shortest path length between node i and node j, and N is the number of nodes in the brain network.

### Statistical Analysis

All statistical analyses were performed in JMP Pro Version 13.2 (SAS Institute Inc., Cary, NC, United States). Linear mixed effects (LME) modeling was performed to test differential changes in EEG power, coherence and global efficiency after iTBS between groups. Group, Timepoint, and Group×Timepoint interaction were included as fixed effects, and subject was included as a random effect. Timepoint was set as repeated covariance structure. Normality of the residuals was visually assessed for each model with conditional residual quantile-quantile plots, and all were found to reasonably conform to the assumption of normality. *Post hoc* tests were performed when *F*-tests were significant. Multiple comparisons between Timepoints or Groups were performed with Tukey-Kramer adjustment.

Data were found to meet the normality assumption using the Kolmogorov-Smirnov test. Pearson correlations were performed to investigate the relationship between baseline and changes in neurophysiological measures (e.g., EEG power, coherence, and global efficiency) and patients characteristics (e.g., age, chronicity, FMA, and ARAT). For all analyses, the statistical significance was set at *P* < 0.05.

## Results

All subjects tolerated iTBS well with no adverse events reported. Individual values of RMT in the ipsilesional hemisphere were presented in Table 2. Of note, RMT was presented as 100% MSO for the individuals in whom MEP was not elicitable in the paretic hand. The averaged RMT in the Active and Sham iTBS groups was 82.7% MSO (SD = 22.3) and 81.5% MSO (SD = 24.9), respectively. The averaged stimulation intensity for iTBS in the Active and Sham iTBS groups was 38.7% MSO (SD = 3.1) and 37.1% MSO (SD = 6.9), respectively. There was no significant difference in RMT or stimulation intensity for iTBS between groups (*P* = 0.900 and 0.457, respectively).

### Electroencephalography Power

The LME modeling did not reveal any significant Group×Timepoint interaction in EEG power in the ipsilesional [*F*_(1,28)_ = 0.02, *P* = 0.893; *F*_(1,28)_ = 1.59, *P* = 0.218; *F*_(1,28)_ = 0.64, *P* = 0.429; *F*_(1,28)_ = 0.70, *P* = 0.409] or contralesional [*F*_(1,28)_ = 0.40, *P* = 0.534; *F*_(1,28)_ = 1.79, *P* = 0.192; *F*_(1,28)_ = 0.10, *P* = 0.753; *F*_(1,28)_ = 0.14, *P* = 0.707] hemisphere in the delta, theta, alpha or beta band, respectively.

### Coherence

**FIGURE 1:**
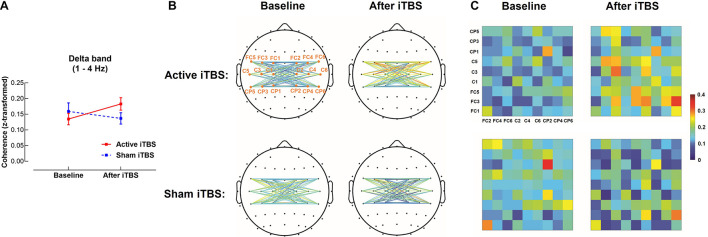
Delta band interhemispheric functional connectivity change after iTBS. Delta band coherence between left and right sensorimotor cortices was significantly increased in the Active iTBS group, but not in the Sham iTBS group after iTBS **(A)**. The topographies **(B)** and matrixes **(C)** represent z-scores of coherences between pairs of electrodes in the left and right sensorimotor cortices before and after iTBS in the Active and Sham iTBS groups. Warmer colors indicate greater coherence, while cooler colors indicate less coherence.

In the delta band, results of LME modeling revealed significant Group×Timepoint interaction [*F*_(1,28)_ = 5.03, *P* = 0.033]. *Post hoc* revealed that in the Active iTBS group, coherence was significantly increased after iTBS compared with baseline (*P* = 0.038), while there was no significant change in coherence over time in the Sham iTBS group (*P* = 0.327) (Figure 1).

**FIGURE 2:**
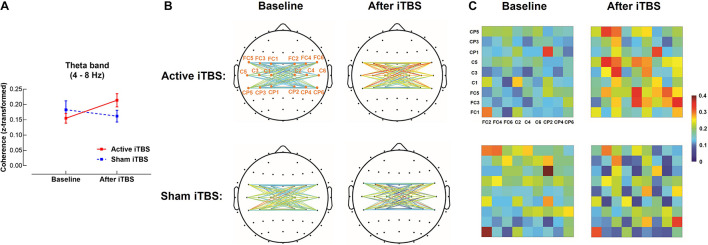
Theta band interhemispheric functional connectivity change after iTBS. Theta band coherence between left and right sensorimotor cortices was significantly increased in the Active iTBS group, but not in the Sham iTBS group after iTBS **(A)**. The topographies **(B)** and matrixes **(C)** represent z-scores of coherences between pairs of electrodes in the left and right sensorimotor cortices before and after iTBS in the Active and Sham iTBS groups. Warmer colors indicate greater coherence, while cooler colors indicate less coherence.

In the theta band, results of LME modeling revealed significant Group×Timepoint interaction [*F*_(1,28)_ = 6.75, *P* = 0.015]. *Post hoc* revealed that in the Active iTBS group, coherence was significantly increased after iTBS compared with baseline (*P* = 0.011), while there was no significant change in coherence over time in the Sham iTBS group (*P* = 0.342) (Figure 2).

**FIGURE 3:**
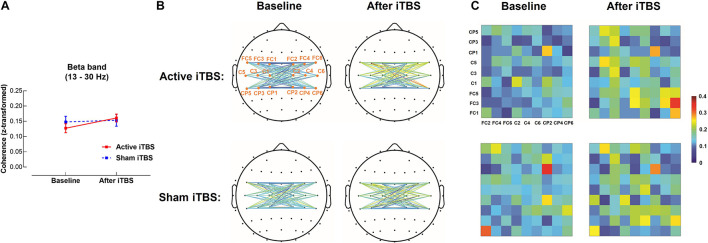
Beta band interhemispheric functional connectivity change after iTBS. Beta band coherence between left and right sensorimotor cortices was significantly increased after Active or Sham iTBS in all subjects without significant group differences **(A)**. The topographies **(B)** and matrixes **(C)** represent z-scores of coherences between pairs of electrodes in the left and right sensorimotor cortices before and after iTBS in the Active and Sham iTBS groups. Warmer colors indicate greater coherence, while cooler colors indicate less coherence.

In the beta band, results of LME modeling revealed significant main effect of Timepoint [*F*_(1,28)_ = 6.38, *P* = 0.018], but there was no significant Group×Timepoint interaction [*F*_(1,28)_ = 3.25, *P* = 0.082], suggesting coherence was increased after iTBS in both groups without group differences (Figure 3).

In the alpha band, the LME modeling did not reveal significant Group×Timepoint interaction [*F*_(1,28)_ = 0.38, *P* = 0.544].

### Global Efficiency

**FIGURE 4:**
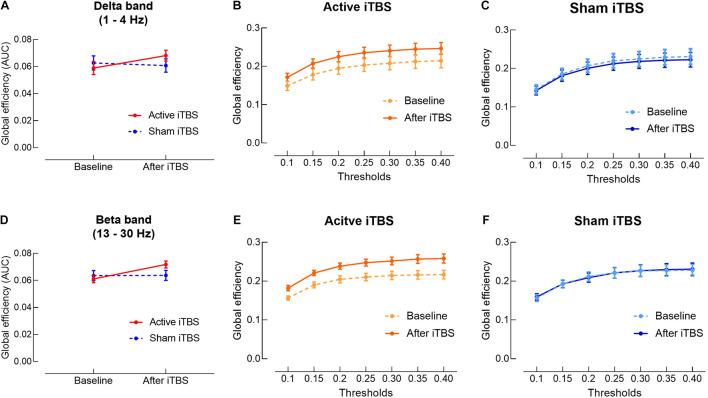
Delta and beta bands global efficiency change after iTBS. Delta **(A)** and beta **(D)** bands global efficiency (area under the curves) were significantly increased in the Active iTBS group, but not in the Sham iTBS group after iTBS. **(B,C)** Delta band global efficiency at each threshold in the Active and Sham iTBS groups, respectively. **(E,F)** Beta band global efficiency at each threshold in the Active and Sham iTBS groups, respectively.

In the delta band, results of LME modeling revealed significant Group×Timepoint interaction [*F*_(1,28)_ = 5.11, *P* = 0.032]. *Post hoc* revealed that in the Active iTBS group, global efficiency was significantly increased after iTBS compared with baseline (*P* = 0.013), while there was no significant change in global efficiency over time in the Sham iTBS group (*P* = 0.586) (Figures 4A–C).

In the beta band, results of LME modeling revealed significant main effect of Timepoint [*F*_(1,28)_ = 8.54, *P* = 0.007] and Group×Timepoint interaction [*F*_(1,28)_ = 8.06, *P* = 0.008]. *Post hoc* revealed that global efficiency was significantly increased after iTBS compared with baseline in the Active iTBS group (*P* < 0.001), while there was no significant change in global efficiency over time in the Sham iTBS group (*P* = 0.954) (Figures 4D–F).

In the theta and alpha band, the LME modeling did not reveal any significant Group×Timepoint interaction [*F*_(1,28)_ = 3.08, *P* = 0.090; *F*_(1,28)_ = 2.19, *P* = 0.150, respectively].

### Correlation Analysis

No significant correlation was observed between neurophysiological measures (i.e., EEG power, coherence and global efficiency) and subject characteristics (i.e., age, chronicity, FMA, and ARAT).

## Discussion

In this study, we measured resting-state EEG at baseline and immediately after iTBS applied over ipsilesional M1 in stroke survivors. To the best of our knowledge, this is the first study that used resting-state EEG to investigate the aftereffects of iTBS in stroke survivors. Our primary findings are: (1) interhemispheric functional connectivity was significantly increased after iTBS; (2) global efficiency was significantly increased after iTBS; and (3) no significant change in EEG power was observed after iTBS.

### Interhemispheric Functional Connectivity

We observed an increase in delta and theta bands coherence between bilateral sensorimotor cortices after iTBS, indicating increased interhemispheric functional connectivity after iTBS. The acute effects of iTBS on functional connectivity have not been investigated in stroke survivors, but it has been investigated in healthy adults (Nettekoven et al., 2014; Hoy et al., 2016). Our results are in line with Hoy et al.’s (2016) study that reported increased interhemispheric functional connectivity in theta band after iTBS in healthy adults. Similarly, an fMRI study (Nettekoven et al., 2014) also reported an increase in functional connectivity between bilateral sensorimotor areas after the application of iTBS on M1 in healthy adults. Despite different methodology among studies, our current study for the first time extends these findings from healthy adults to stroke population, suggesting that iTBS produces similar effects on interhemispheric functional connectivity in stroke survivors and healthy adults.

Neural mechanisms underlying the increase in interhemispheric functional connectivity after iTBS in stroke survivors remain unclear, which possibly relates to the simultaneous induction of neural activity in the whole motor network during the application of iTBS (Nettekoven et al., 2014). It has been reported that rTMS-induced changes in cortical activity are not exclusively local, but also extending to remote, interconnected regions (Bestmann et al., 2004; Suppa et al., 2008; Cardenas-Morales et al., 2014). As bilateral sensorimotor cortices are interconnected by transcallosal fibers, iTBS applied on ipsilesional M1 would induce simultaneous activation in the contralesional sensorimotor cortex (Nettekoven et al., 2014). The simultaneous activation of bilateral sensorimotor cortices would contribute to an increase in the coherence of brain activity that represents an important neurophysiological mechanism enforcing communication between the interconnected brain regions via transcallosal connections, and thus increases interhemispheric functional connectivity (Fries, 2005; Di Lazzaro et al., 2008).

Interestingly, beta band coherence was increased in both Active and Sham iTBS groups without significant group difference, suggesting changes in beta band coherence might not relate to neural effects of iTBS but result from other confounding factors such as the noise of iTBS click (Fuggetta et al., 2008). An increase in beta band coherence was also reported after sham rTMS in healthy adults (Fuggetta et al., 2008). The increased beta band coherence after sham rTMS may be caused by a cumulative effect of the rapid sequency of auditory TMS-click sounds produced during the application of rTMS (Fuggetta et al., 2008). Some neuroimaging studies (Bestmann et al., 2004; Takano et al., 2004) suggested that TMS clicks induce activations of the auditory systems and influence cerebral blood flow and synaptic activity in the brain regions interconnected with the auditory systems, which possibly influences beta band coherence between bilateral sensorimotor cortices. Collectively, external influences on cortical oscillations due to concomitant auditory stimulation need to be carefully controlled in TMS studies (Fuggetta et al., 2008).

### Global Efficiency

We observed an increase in delta and beta bands global efficiency after iTBS. Acute changes in global efficiency induced by iTBS or high frequency rTMS have not been investigated in stroke survivors. Park et al. (2014) investigated acute changes in global efficiency after high frequency rTMS in healthy adults. No significant change in global efficiency was observed in the whole sample, but the authors reported an increase in global efficiency in individuals with behavioral facilitation after rTMS (Park et al., 2014). In our current study, we observed an increase in global efficiency after iTBS in the whole sample. The inconsistent results between Park et al.’s (2014) and our study may result from differences in subjects’ characteristics (i.e., healthy adults in Park et al.’s (2014) study vs. stroke survivors in ours) and experimental methodology. For example, Park et al. (2014) used 10 Hz rTMS, while we used iTBS in the present study. As the neural effects produced by iTBS have been suggested to be stronger than traditional rTMS (Fuggetta et al., 2008), it is reasonable to speculate that increase in global efficiency is possibly more robust after iTBS compared with 10 Hz rTMS.

The mechanisms for the increase in global efficiency after iTBS has not been fully elucidated. It has been suggested that iTBS induces long-term potentiation (LTP)-like changes at synaptic connections (Huang et al., 2007), and would increase efficiency of synaptic transmission in both local and remote brain regions from the stimulation site (Philips et al., 2017). Furthermore, iTBS causes simultaneous activation in the interconnected brain regions which increases neural synchrony in the global brain network (Di Lazzaro et al., 2008). Therefore, iTBS possibly facilitates global information exchange and thus increases global efficiency.

### Electroencephalography Power

We did not observe any change in EEG power after iTBS. Although there was no stroke study investigating aftereffects of rTMS (including iTBS) on EEG power, our results are in line with studies conducted in healthy adults, which reported no change in EEG power after iTBS (Hoy et al., 2016) or high frequency rTMS (Oliviero et al., 2003; Fuggetta et al., 2008); however, increased EEG power after high frequency rTMS has also been reported (Azila Noh and Fuggetta, 2012). These conflicting results may be due to the different methodological details among studies. For example, 10 Hz rTMS was applied in Azila Noh and Fuggetta (2012) study, while 5 Hz rTMS was applied in Oliviero et al.’s (2003) and Fuggetta et al.’s (2008) studies. These results suggest that different types of rTMS might influence its effect on EEG power.

### Clinical Implications

This study for the first time used EEG to investigate the aftereffects of iTBS following stroke. Our results revealed increased interhemispheric functional connectivity and global efficiency after iTBS in stroke survivors. Dynamics of functional brain network has been suggested to be altered following stroke due to focal brain lesions (Vecchio et al., 2019). Normal functioning of brain network (i.e., interhemispheric functional connectivity and global efficiency) plays an important role in recovery of motor performance following stroke.

Interhemispheric functional connectivity has been suggested to play a supportive role in motor recovery following stroke (Rehme et al., 2011). van Meer et al. (2010) reported that impaired motor function acutely after experimental stroke in rats was related to partial loss of interhemispheric functional connectivity, and interhemispheric functional connectivity was increased subsequently concomitant to motor recovery. In humans, reduced interhemispheric functional connectivity was also observed acutely after stroke (Philips et al., 2017; Desowska and Turner, 2019; Hoshino et al., 2020; Li et al., 2020). It has been reported that the increase in interhemispheric functional connectivity was associated with motor improvements in stroke survivors, and restoration of interhemispheric functional connectivity was noted only in well recovered individuals, but not in the poorly recovered stroke survivors (Golestani et al., 2013; Desowska and Turner, 2019; Hoshino et al., 2020; Li et al., 2020), suggesting that interhemispheric functional connectivity is a potential biomarker indicating stroke recovery (Caliandro et al., 2017; Philips et al., 2017; Vecchio et al., 2019). Increase in interhemispheric functional connectivity after iTBS observed in our current study provides evidence that iTBS could normalize brain network functioning in stroke survivors.

Global efficiency exhibits the efficiency in transporting information at a global scale between genetic brain areas (Vecchio et al., 2019). Increased global efficiency after iTBS suggests alterations in how efficiently information is transferred over the brain, reflecting an acute shift of the brain state induced by iTBS (Park et al., 2014). Reduced global efficiency indicates lower efficiency in global information flow, which has been suggested to be related to motor deficits associated with aging (Park et al., 2012) or neurological conditions such as stroke (Philips et al., 2017). Contrary to the brain state of motor deficits, increased efficiency in global information flow could reflect the brain state of intact or enhanced motor function (Park et al., 2014). Therefore, the shift of brain state toward an emphasis on global information exchange after iTBS suggests that iTBS has the potential to be utilized in stroke rehabilitation.

The influence of stroke characteristics (e.g., chronicity, motor impairment, age, etc.) on the effects of iTBS is less clear. In present study, no significant correlation observed between clinical characteristics and neurophysiological measures was observed. Our results suggest that the effects of iTBS on functional brain network were not influenced by stroke characteristics. As our sample size is small (*N* = 30), cautions are needed when interpreting these results. Further studies with larger sample sizes are still needed to investigate how the heterogeneity of stroke survivors influences the effects of iTBS.

### Limitations

As a pilot study, the sample size of current study is small (*N* = 30). Chronicity of stroke survivors in current study ranged from 1 to 18 months, so cautions are needed when generalizing our findings to more chronic stroke survivors. Chronicity of stroke survivors was not evenly distributed in our sample, and most subjects were within 3 months post-stroke. Therefore, we did not perform subgroup analysis for chronicity. Further research is required to test our results in stroke survivors with a wider range of chronicity with larger sample sizes and to perform subgroup analysis for individuals in acute, subacute and chronic phases of stroke.

Our current study only measured resting-state EEG for 6 min immediately after iTBS without a follow-up. We understand that it would be more meaningful to measure EEG at multiple time points after iTBS. However, it has already been a long experiment for stroke survivors, and many subjects could not tolerate for a longer time of data collection. Further studies are needed to monitor changes in EEG at multiple time points after iTBS.

Another limitation is that sex of stroke survivors was not very balanced between groups with 12 males in the Active iTBS group vs. 9 males in the Sham iTBS group. To the best of our knowledge, no previous study has reported sex difference in the aftereffects of TBS. There was a tDCS study (Kuo et al., 2006) reporting sex differences in the aftereffects of cathodal (i.e., inhibitory) but not anodal (i.e., excitatory) tDCS due to changes in ovarian hormones over the menstrual cycle. In current study, most female subjects (8 out of 9) were postmenopausal women. Those postmenopausal women did not have a menstrual cycle, so they were less likely to be influenced by changes in ovarian hormones. Although it is still unclear whether sex influences aftereffects of iTBS, our results are unlikely to be attributed to sex difference. Further studies are still needed to investigate sex difference in the aftereffects of iTBS.

In this study, 40% MSO was the upper limit for iTBS with the TMS machine. We set the stimulation intensity of iTBS at 40% MSO if the calculated stimulation intensity (i.e., 70% RMT) was greater than 40% MSO. Therefore, the actual stimulation intensity of iTBS was lower than the calculated stimulation intensity for those whose RMT was greater than 57% MSO. In 11 out of 15 subjects in the Active iTBS group, RMT was greater than 57% MSO. We acknowledge that the relatively lower stimulation intensity of iTBS for subjects with high RMT is a limitation of current study. However, as higher stimulation intensity may produce stronger neurophysiological effects, neuroplastic changes observed in current study were induced by relatively lower stimulation intensity, suggesting that the neuroplastic changes observed in current study were robust.

### Conclusion

Ours is the first study that used EEG to investigate the aftereffects of iTBS on functional brain network in stroke survivors. This study for the first time provides evidence that iTBS modulates functional brain network in stroke survivors. Our results revealed an increase in interhemispheric functional connectivity and global efficiency after iTBS, suggesting that iTBS has the potential to normalize brain network functioning following stroke, which can be utilized in stroke rehabilitation.

## Data Availability Statement

The raw data supporting the conclusions of this article will be made available by the authors, without undue reservation.

## Ethics Statement

The studies involving human participants were reviewed and approved by the Guangzhou First People’s Hospital Human Research Ethics Committee. The patients/participants provided their written informed consent to participate in this study.

## Author Contributions

YL, GX, and QD designed the experiment. JiC and YP recruited the participants. QD, SZ, SC, XL, JuC, YC, and KC conducted the experiments. QD, SZ, GC, and JiC reduced and analyzed the data. QD and SZ interpreted the data. QD, SZ, and YL wrote the manuscript. All authors contributed to the article and approved the submitted version.

## Conflict of Interest

The authors declare that the research was conducted in the absence of any commercial or financial relationships that could be construed as a potential conflict of interest.

## Publisher’s Note

All claims expressed in this article are solely those of the authors and do not necessarily represent those of their affiliated organizations, or those of the publisher, the editors and the reviewers. Any product that may be evaluated in this article, or claim that may be made by its manufacturer, is not guaranteed or endorsed by the publisher.

## Abbreviations

rTMS: repetitive transcranial magnetic stimulation
iTBS: intermittent theta burst stimulation
MEP: motor evoked potential
M1: primary motor cortex
EEG: electroencephalography
fMRI: functional magnetic resonance imaging
FMA: Fugl-Meyer assessment
ARAT: action research arm test
IH: ipsilesional hemisphere
RMT: resting motor threshold
EMG: electromyography
FDI: first dorsal interosseous
MSO: maximum stimulator output
ICA: independent component analysis
AUC: area under the curve
LME: linear mixed effects
LTP: long-term potentiation.
